# Retraction notice for: “Effect of lncRNA HULC knockdown on rat secreting pituitary adenoma GH3 cells” [Braz J Med Biol Res (2019) 52(4): e7728]

**DOI:** 10.1590/1414-431X20207728retraction

**Published:** 2020-08-03

Qiu Hong Ruihttps://orcid.org/0000-0002-5459-0259^1^, Jian Bo Mahttps://orcid.org/0000-0003-4645-860X^1^, Yu Feng Liaohttps://orcid.org/0000-0002-6402-5405^1^, Jin Hua Daihttps://orcid.org/0000-0002-2490-9636^1^, and Zhen Yu Caihttps://orcid.org/0000-0002-8364-3954^2^

^1^Department of Clinical Laboratory, HwaMei Hospital, University of Chinese Academy of Sciences (Ningbo No. 2 Hospital), Ningbo, Zhejiang, China

^2^Department of Pain Clinic, The First Affiliated Hospital of Xiamen University, Fujian Medical University, Xiamen, Fujian, China

Correspondence: Jin Hua Dai: <daijinhua418@sina.com> | Zhen Yu Cai: <fangpoji7015mad@163.com>

**Retraction for:** Braz J Med Biol Res | doi: http://10.1590/1414-431X20197728 | PMID: 30994730 | PMCID: PMC6472935

The Brazilian Journal of Medical and Biological Research received a request from the authors to withdraw this manuscript. Meanwhile, the Editors became aware of a denouncement published by independent journalists from the “For Better Science” website including this paper. This denouncement consisted of potential data falsification and/or inaccuracy of results in western blots and flow cytometry plots.

As per consensus between the Authors and the Editors-in-Chief of the Brazilian Journal of Medical and Biological Research (BJMBR), the article titled “Effect of lncRNA HULC knockdown on rat secreting pituitary adenoma GH3 cells” that was published in year 2019, volume 52, issue 4, (Epub Apr 15, 2019) has been retracted.

